# Teaching the laboratory assessment of neutrophil chemotaxis: a simulation-based approach for undergraduate immunology

**DOI:** 10.1093/immhor/vlaf049

**Published:** 2025-10-09

**Authors:** Maurizio Costabile, Gareth Denyer

**Affiliations:** University of South Australia, Clinical and Health Sciences, Adelaide, South Australia, Australia; School of Life and Environmental Sciences, University of Sydney, Sydney, New South Wales, Australia

**Keywords:** chemotaxis, education, neutrophil, simulation, undergraduate

## Abstract

Neutrophils are essential cellular components of innate immunity. After injury, they migrate into tissues following chemotactic gradients to phagocytose pathogens or respond to tissue damage. This multistep process is tightly regulated, and defects at any stage can lead to increased bacterial infections. Identifying specific defects requires specialized assays, yet teaching the assessment of these functions in a laboratory setting presents challenges. At the University of South Australia, undergraduate immunology is taught to students training as laboratory medicine scientists, who must understand how to assess neutrophil function. However, demonstrating chemotaxis in the laboratory is not possible due to a lack of inverted microscopes, restricted laboratory time, and lack of patient samples with defined neutrophil defects. To address this, we developed a computer simulation replicating the under-agarose method of quantifying neutrophil chemotaxis. In the simulation, students load both “control” and “patient” samples and measure both random and directed migration toward 5 common chemoattractants. Using an in-house–defined reference range, they determine the immunological status of each sample. The simulation’s impact was evaluated using a mixed-methods approach, incorporating Likert-scale questionnaires, free-text feedback, and scores from laboratory reports. Student feedback was overwhelmingly positive, with the simulation significantly enhancing their understanding of neutrophil function. All students successfully completed the report, typically achieving high grades. These findings support the use of authentic computer-based simulations as effective alternatives for teaching complex immunological techniques in resource-limited settings, offering a practical and engaging solution to challenges in traditional laboratory instruction.

## Introduction

Neutrophils are a key cellular component of innate immunity.^1^ Following systemic injury, neutrophils are the first cells to migrate from the blood into tissues following a chemotactic gradient to phagocytose pathogens or respond to tissue damage.^2^ This complex process requires several well-controlled events, and defects at any stage, such as adhesion, diapedesis, migration, and phagocytosis, present clinically as an immunodeficiency disease typically associated with increased bacterial infections.^3^ Assessing potential defects at each of these stages requires specialist assays, reagents, and equipment.^4^ As a result, teaching the laboratory assessment of each of these important stages at an undergraduate level can be challenging. At the University of South Australia (UniSA), Laboratory Medicine students constitute the largest undergraduate cohort taught immunology, in preparation for a career in a clinical diagnostic environment. Thus, they must have a thorough understanding of how individual defects in neutrophil function can be assessed. We cannot demonstrate neutrophil chemotaxis in a face-to-face laboratory setting due to a lack of inverted microscopes, laboratory time limitations, and importantly, access to patient cells with a defined neutrophil chemotactic defect. Even if we chose to use normal healthy donor cells, factors such as the requirement for freshly isolated cells, the condition of the neutrophils, the chemoattractants chosen, and the precision of the experimental setup can lead to inconsistent outcomes. Last, the complexity and length of chemotaxis experiments could result in students spending a significant amount of time preparing reagents rather than actively learning about the cellular mechanisms behind chemotaxis. Given these challenges, we chose to teach this process through computer simulations or a virtual laboratory.

Computer simulations or virtual laboratories have become a well-established and effective pedagogical strategy in higher education, particularly in science disciplines. They can be valuable alternatives or supplements to traditional methods, allowing students to engage with the process in a more interactive and less constrained environment where practical laboratory work is constrained by logistical, financial, or safety considerations.^5–7^ Virtual laboratories enable the visualization of complex, dynamic, or microscopic processes that are otherwise difficult to observe, such as cellular migration.^8^^,^^9^ Furthermore, they provide a safe environment for students to practice, repeat experiments, and learn from mistakes without the consequences of resource consumption or experimental failure, fostering a deeper conceptual understanding.^10^^,^^11^ This approach aligns with constructivist learning theories, where students actively build knowledge by manipulating variables and observing outcomes. Simulations have been successfully implemented across various fields, including genetics,^12^ physiology,^13^ and clinical diagnostics,^14^ to bridge the gap between theoretical knowledge and practical application, demonstrating their value in preparing students for real-world scenarios.^15^^,^^16^

To address this, we developed an authentic computer simulation that replicated the under-agarose method of quantifying neutrophil migration.^17^^,^^18^ In this assay, neutrophils are placed into a well in the center of an agarose plate. To 1 side is added the diluent, while to a well on the other side is added a chemoattractant, such as phorbol myristate acetate (PMA).^19^ Normal neutrophils will sense the chemotactic gradient and begin to migrate towards the well. After a period of incubation, the distance travelled is measured using an eyepiece graticule and compared with normal reference ranges. Migration towards the diluent is used to measure random migration. Due to the limitations outlined above in providing hands-on experience in this assay, our primary research question was to evaluate whether a computer-based simulation could effectively support undergraduate learning of neutrophil chemotaxis, offering a pedagogically sound alternative to traditional laboratory instruction in a resource-limited setting.

Learning objectives Upon completion of the online chemotaxis simulation, students will be able to:

1. Define neutrophil chemotaxis and describe the basic features of the under-agarose method

2. Identify common chemoattractants used in a clinical laboratory

3. Differentiate between the concepts of random and directed cell migration

4. Explain how cellular defects can manifest as reduced levels of neutrophil migration

5. Analyze and interpret quantitative data generated from a simulated chemotaxis assay

6. Evaluate the migration status of simulated patient samples to determine potential immunological causes for reduced chemotaxis

## Materials and methods

### Student cohort

In 2024, 70 undergraduate students were enrolled in Immunology (BIOL 2037) at UniSA, a core second-year course for all students enrolled in Laboratory Medicine and Biomedical Science, and an elective for students enrolled in Pharmaceutical Science and Science programs. Students completed the online chemotaxis simulation in week 7 (of 13) of the semester with teaching staff present. Students were provided with detailed documentation (see Supplementary material) to undertake all the required steps.

### Virtual chemotaxis

The online hemocytometer was created using the Unity game engine and can be accessed at https://garethdenyer.github.io/Chemotaxis/. In creating the simulation, we took advantage of universal learning design.^20^^,^^21^ This framework ensures that the needs and abilities of all learners are accommodated in a blended learning environment and eliminates unnecessary hurdles in learning. The study was conducted in a university computer pool, where students individually worked through the self-guided online simulation using the provided written documentation.

### Simulation development

**Figure 1.**
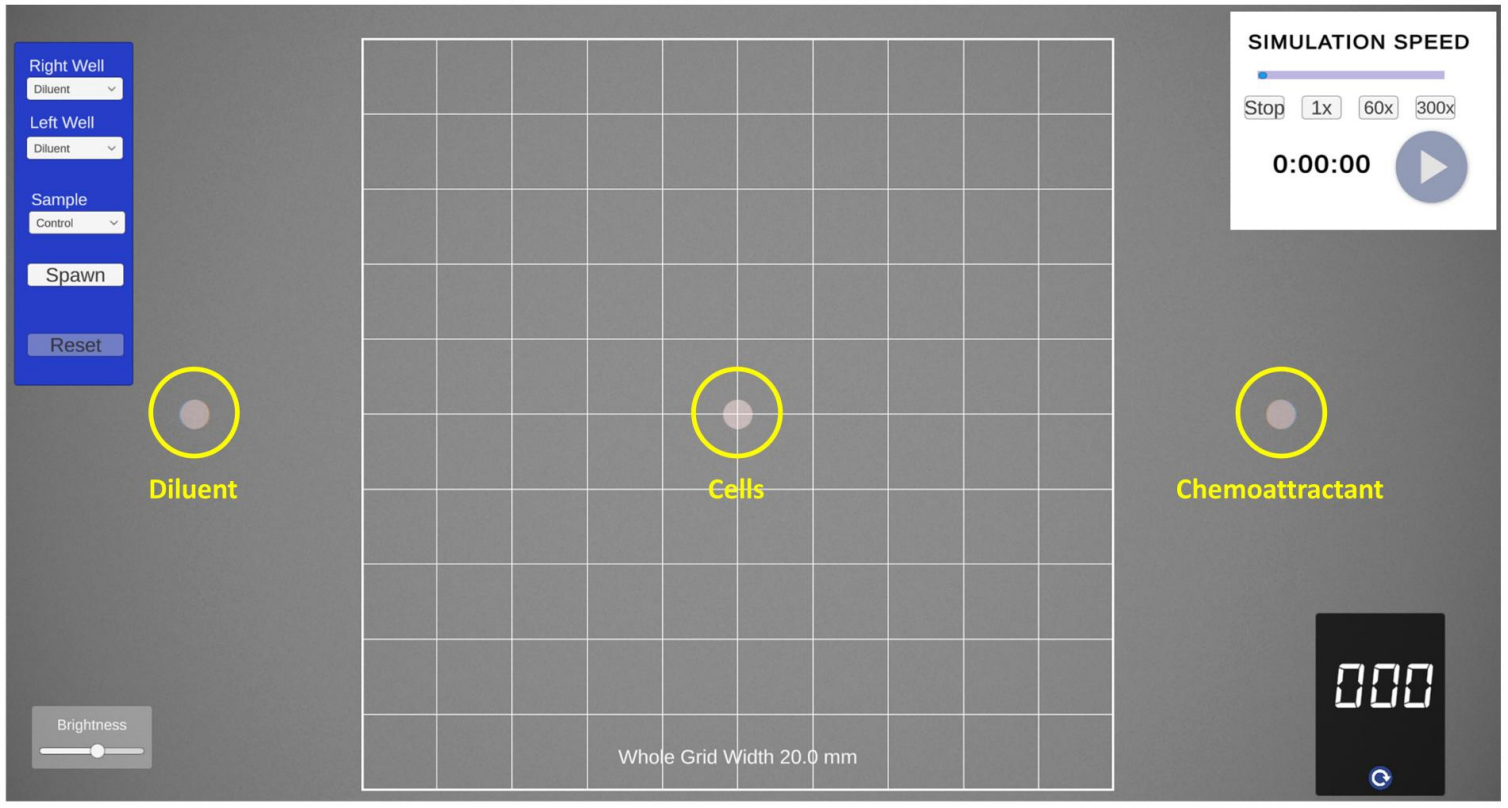
View of the chemotaxis simulation on loading. Moving from left to right, the yellow circles indicate the wells where the diluent is loaded, cells are loaded and the chemoattractant is loaded. These locations can be changed, but the cells must always be in the central well. The timer (top right) controls the start of the simulation. Students press the start arrow and can then accelerate time and observe the cells migrating towards the diluent and chemoattractant, respectively.

**Figure 2.**
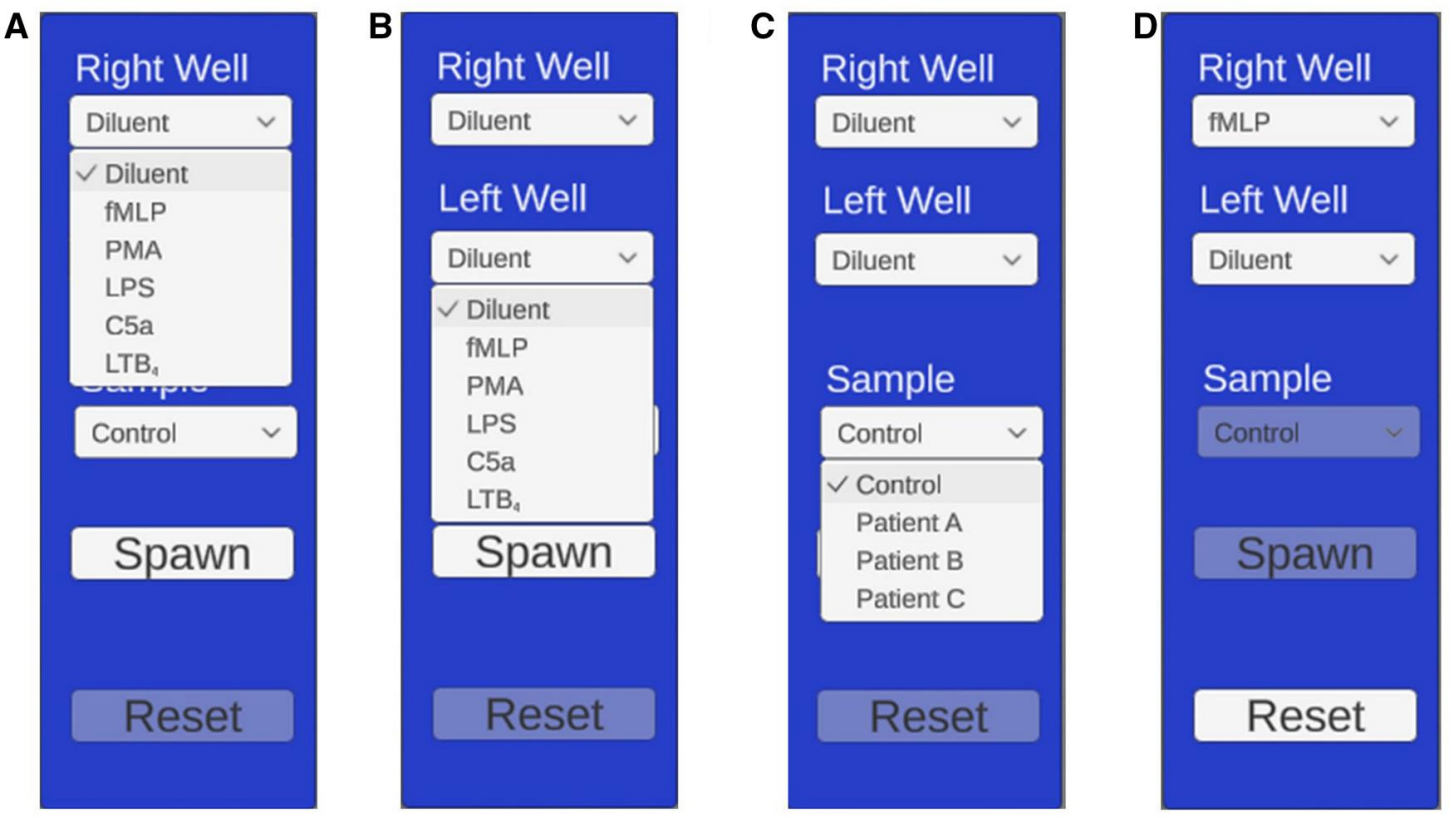
View of the available options in the pull-down menus. (A) List of reagents that can be added to the right-hand well. (B) List of reagents that can be added to the left-hand well. (C) Shows the options for control or patient samples that can be added to the central well. (D) The “Spawn” button loads all samples into the well, while the “Reset” button changes all parameters back to their basal state.

**Figure 3.**
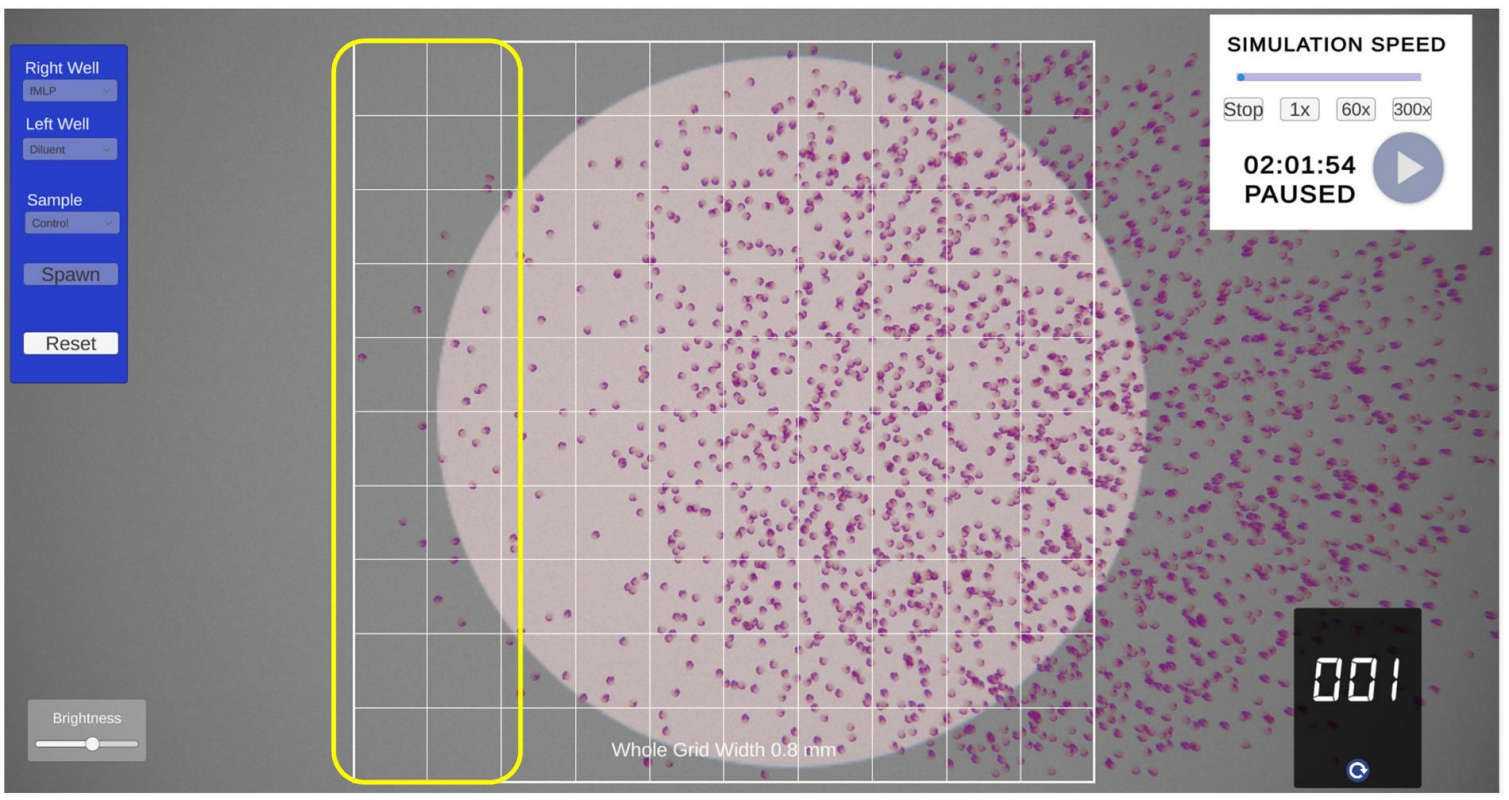
Magnified view of cells that migrated towards the diluent (random migration). The distance migrated can be measured using the overlaid 10 unit-wide grid; the total width shown is 0.8 mm, indicating each grid represents 0.08-mm distance. The yellow rectangle highlights the migrated cell population.

**Figure 4.**
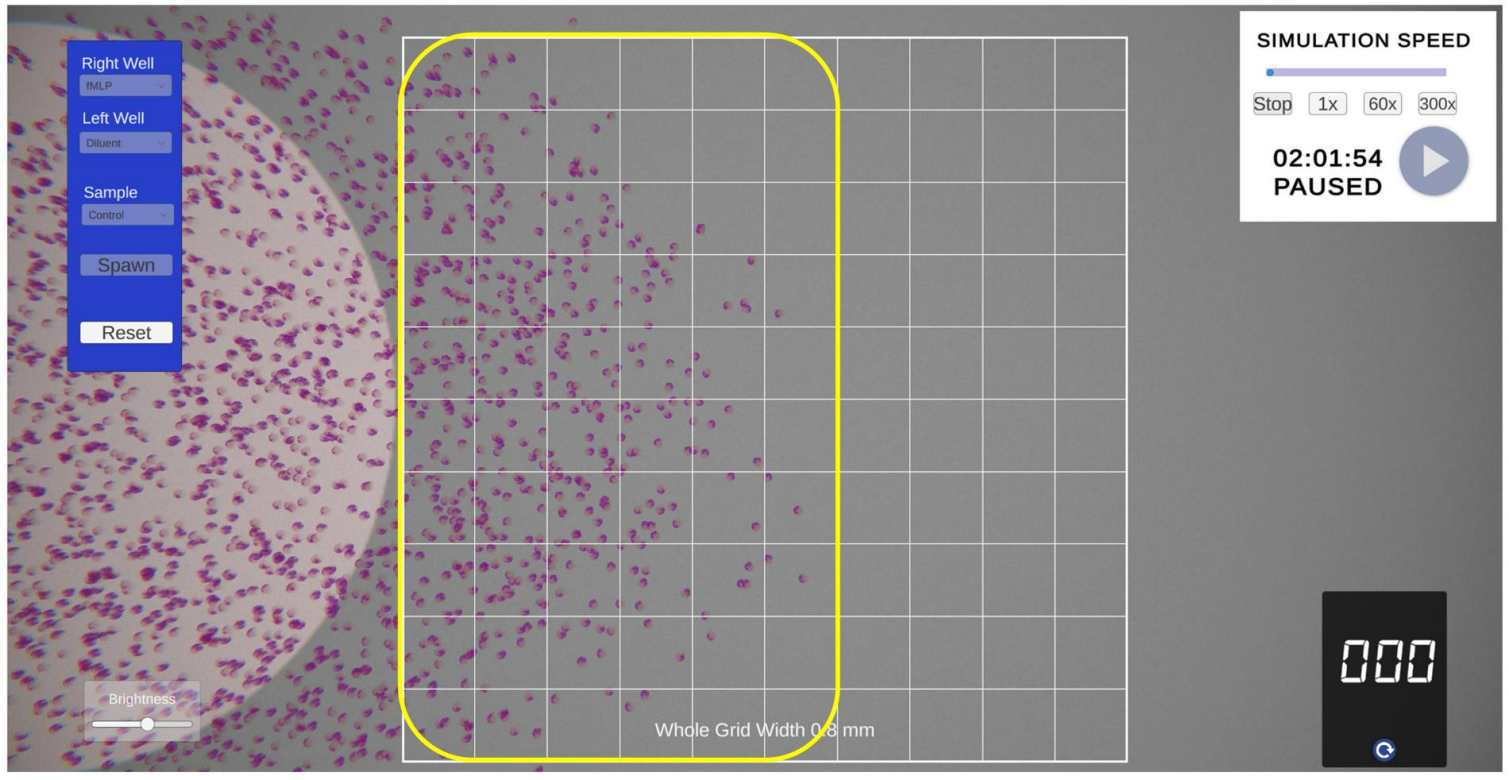
Magnified view of cells that migrated towards a chemoattractant (directed migration). The distance migrated can be measured using the overlaid 10-wide grid; the total width shown is 0.8 mm, indicating each grid represents 0.08-mm distance. The yellow rectangle highlights the migrated cell population, which equates to 5.5 grids and hence a total distance of 0.44 mm.

The simulation replicated the under-agarose gel method^17^ providing a microscopic view of the wells, cells, and grid for determining migration distances. On startup, a top-down view of a plate filled with agar and 3 evenly spaced wells is visible with a central overlaid 10 × 10 grid (Fig. 1). This grid is used to measure the distance cells have migrated from the central well following a 2-h “virtual” incubation. The student can specify which reagent is added to either the left or right well, and which cells (control or patient samples) are loaded into the central well (Fig. 2). The reagents include diluent or common chemoattractants, including fMLP,^22^ PMA,^23^ LPS,^24^ complement component 5a (C5a),^25^ or leukotriene B_4_ (LTB_4_)^26^ (Fig. 2). Once the wells are loaded, the simulation can begin. To account for real-world time limitations, the incubation time can be accelerated to varying levels (1, 60, or 300×) (Fig. 3). The cells can be actively visualized migrating either towards the diluent (random migration) or towards the chemoattractant (directed migration) (Fig. 4). After 2 h of virtual migration, the distance travelled from the central well is measured using the 10 × 10 grid. On zooming, the size of the grid is adjusted allowing for fine measurements of distance travelled. Cells that travel further than the grid can be selected using the mouse, turning green, and are then used as a reference point for moving the grid. This process allows for accurate measurement of the total distance travelled.

### Student assessment: the laboratory report

Student performance in the practical report and written feedback was used to evaluate the effectiveness of the simulation. To assess learning, the laboratory report tasked students with analyzing simulated data from 1 control and 2 patient samples. First, students measured the migration distance (mm) for both random and directed chemotaxis. Next, they interpreted these results by comparing them with a provided reference range, classifying each sample as either normal or indicative of a potential immunological defect. Finally, the laboratory report included 5 short-answer questions to evaluate students’ understanding of core theoretical concepts. The full laboratory report and grading rubric are available in the Supplementary materials. The rubric addressed the ability of students to correctly determine the validity of the control cell migration, patient migration, use of the reference range, and ability to answer the study questions.

### Questionnaire design and validation

A custom questionnaire based on similar prior studies^6^^,^^27^^,^^28^ was developed to evaluate the effectiveness and usability of the chemotaxis computer simulation. The questionnaire (see Supplementary material) consisted of 12 items, including 8 quantitative items (seven 5-point Likert scales and one 3-point question) and 4 open-ended qualitative questions. The quantitative questions (Q) were designed to assess 4 distinct constructs: Q1–Q4 examined ease of use, clarity of instructions, and graphics; Q5 and Q6 examined learning gains using a pre-test and post-test pair of questions to measure self-reported understanding of chemotaxis before and after the simulation; Q7 assessed changes in student interest with the content; Q8 assessed student confidence in applying the concepts; and Q9 examined the likelihood of recommending the simulation to other students. The internal consistency for Q1–Q4 was assessed to ensure its reliability. The analysis yielded a Cronbach’s α of 0.88, indicating a high level of internal consistency and supporting the reliability of these questions.^29^

### Simulation implementation

The student cohort was divided into 3 separate classes that each undertook 1 online session per week. For the simulation, students undertook the simulation in a university computer pool (20 students/room). Detailed instructions outlining all the steps were provided to students in their practical manual (see Supplementary material). Each student was allocated a normal control and 2 patient samples. For each of the samples, 1 of the 5 chemoattractants was tested at a single concentration. Cells were incubated for 2 h (accelerated time), and both random and directional migration were measured in millimeters. These results were tabulated and, on completion, compared with a provided reference range where the student could determine if the control was normal, if defects were identified, and the immunological status of the patient samples.

### Student feedback

Following the laboratory session, students completed a questionnaire containing a combination of Likert scale and free-text responses (Supplementary materials). Results were scored as 1, strongly disagree; 2, disagree; 3, neutral; 4, agree; and 5, strongly agree. Three free-text questions relating to the best aspects, most challenging aspects of the simulation, and suggestions for improvement were also included and analyzed via thematic analysis.^30^

### Statistical analysis of results

The responses for the Likert-style questions were scored as above and statistics calculated using GraphPad Prism (version 10.0.3). For 5-point Likert items, either parametric *t* tests or nonparametric Mann-Whitney tests are suitable to assess changes in responses.^31^ Results were analyzed using Student’s *t* test. A *p*-value < 0.05 was considered statistically significant.

### Ethical clearance

Ethical clearance for this study was granted by the UniSA Human Research Ethics Committee (#204873).

## Results

### Student demographics

In 2024, the demographic data were as follows: *n* = 70; mean ± SD age, 23.69 ± 6.12 years (range, 19.5 to 56 years); 51 females (73%); and 19 males (27%). Fifty students (71.4%) were enrolled in Laboratory Medicine, 14 (20%) in Biomedical Science, 4 (5.7%) in Pharmaceutical Science, and 2 (2.8%) in the Bachelor of Science. The majority, 64 (91.4%), were local and 6 (8.6%) were international students. Only 10 students were enrolled part-time (14%) and the remainder were enrolled full-time (86%). The mean ± SD GPA (grade point average) for the students was 4.98 ± 1.1 (maximum of 7).

### Student assessment: laboratory report

Immunology (BIOL 2037) includes 6 practicals (repeated over 2 wk), which, due to limited available laboratory space, are completed over the 13-wk semester. Of these 6 practicals, each student is allocated 2 reports for formal assessment, while the others receive formative feedback. All students are required to submit a report irrespective of whether it is marked or only receives formative feedback. Students were given 1 wk from the date of the laboratory simulation to complete and submit the written report. Thus, 24 of the 70 students completed a formal report for assessment. Although only 24 reports were submitted for formal marking, all other students completed the report for formative feedback. Hence, we were able to assess the responses to the 5 study questions that delve deeper into the students’ understanding of the core concepts being demonstrated in the simulation. Using the marking rubric, a maximum total score was 40 marks. All students who submitted a formal report for assessment passed, with the mean score being 30.46 ± 4.16 (76%), which equates to a distinction grade. This result indicated very good performance in the written report. The individual score distribution for these students can be viewed in Fig. S1. As reference to the typical score achieved for a formal report by this cohort, the mean score for an F-to-F laboratory assessment was 32.95 ± 6.6 (maximum of 48), which is a score of 68%. Hence, there was no significant difference (Student’s *t* test, *p *= 0.75) in student performance in both an F-to-F laboratory or online simulation.

#### Analysis of laboratory report study question responses

The following questions were included in the laboratory report aimed at gauging students’ understanding of key concepts, data generated, and their interpretation of the generated results.

*Q1. If cells taken from the normal control migrated less that the normal reference range, what would this mean? Could you interpret the patient results? Explain your answer.*

This question was aimed at assessing the students’ understanding of the importance of a normal control and how this affects the ability to interpret a patient result. All students have previously completed Biochemistry, Physiology, and Microbiology in the first semester of the second year. In these courses, the importance of a control sample and how it affects the validity of test results is discussed in detail. Hence, students should be highly familiar with this important concept and be able to respond appropriately. Indeed, 95% of students correctly identified that, if the normal control cells migrated less than the reference range, the results were invalid, and the immune status of the patient cells could not be determined. Students included several valid reasons for potentially abnormal results, including degraded chemoattractants, incorrect concentrations of chemoattractants being used, and variations in assay parameters, such as incubation temperature, demonstrating a good understanding of parameters key to assay reliability.

*Q2. Considering the results for the normal control, discuss the results collected for the 2 patient samples.*

The simulation was designed so that the patient samples generated results below the normal reference range as this would then stimulate a better understanding of the potential mechanism(s) of action for the defects. In some instances, the simulation algorithm may have also generated patient results that were slightly above the reference range, yet these are still valid as the focus of the exercise was to observe a deficiency in migration.

*Q3. If the patient cells were found to migrate less than the reference range, give 1 immunological explanation for this result.*

This question required students to proactively search for information, as this content was yet to be covered in lectures. Hints were given to students regarding which upcoming lectures could be of assistance. Perhaps expectedly, there was much variation in responses to this question. Students correctly identified that patients with leukocyte adhesion deficiency^32^ would be captured using this assay. They also discussed the potential of cells having defects in relevant chemoattractant receptor expression (eg, fMLP).^33^ However, since patient samples were typically defective to 2 different agonists, this is unlikely to be the case for 2 different chemoattractants. In some cases, students incorrectly suggested that IL-8 levels may have been reduced. This reference came from a lecture on chemokines and inflammation where the role of IL-8 in vivo leads to neutrophil activation.^34^ Here, there was confusion between an in vitro assay vs the in vivo setting. Last, there was 1 other incorrect response related to HIV affecting chemokine receptor expression (CXCR4), again as this was covered in the chemokine lecture.

*Q4. Briefly explain the nature of the 2 chemoattractants assigned to you and why they are suitable to be used in this assay.*

This question required the most research, particularly for fMLP, PMA, and LTB_4_, which are not covered in lecture content. In most cases, this question was answered well and with appropriate journal references included. This question should be relatively easy to complete as there is ample information present in the literature.

*Q5. What technical errors might occur with this assay? How would they be identified?*

This was a more technically challenging question as the students were unable to complete any of these aspects in a F-to-F setting. However, given the authentic visual representation of the simulation, it did allow them the opportunity to visualize the process completed online and predict potential issues given their knowledge of immune cells. They, again, identified issues with cell preparation and viability affecting the assay result, and the running of controls was key to identifying these issues.

#### Student questionnaire feedback

**Figure 5.**
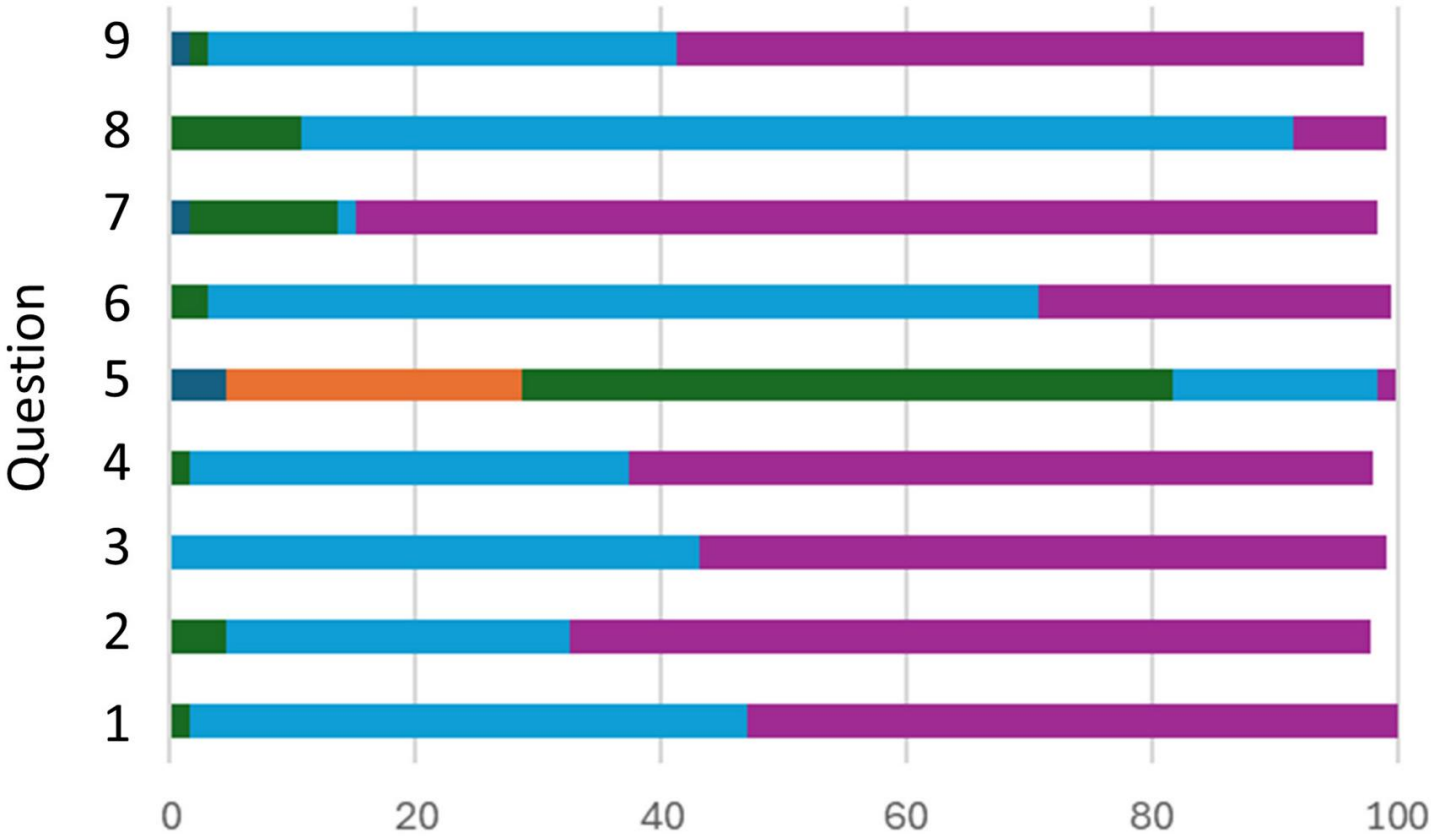
Graphical distribution of student responses to the Likert questions. Strongly agree (Purple); Agree (Light blue); Neutral (Green); Disagree (Orange); Strongly disagree (Dark blue).

Student feedback on the simulation’s design, usability, and impact was assessed using seven 5-point Likert scale questions. The distribution of student responses for each item is shown in Fig. 5, and a summary of the descriptive statistics is presented in Table S3. Student feedback was exceptionally positive across all assessed domains. The items related to the simulation’s usability and clarity (Q1–Q4) showed a strong positive skew, with mean scores ranging from 4.5 to 4.6. Notably, no students selected “Disagree” or “Strongly Disagree” for any of these 4 items, indicating a universally positive perception of the simulation’s design and instructions. Prior to the simulation (Q5), responses were distributed across the scale (mean = 2.8), reflecting a baseline of poor-to-neutral understanding. In contrast, post-simulation responses (Q6) cluster at the high end of the scale (mean = 4.2), demonstrating the significant improvement in understanding (Student’s *t* test, *p *< 0.0001). Finally, students reported strong confidence in applying the concepts (Q8, mean = 3.9) and overwhelmingly indicated they would recommend the simulation to their peers (Q9, mean = 4.5). Collectively, these results provide robust graphical evidence that students found the simulation to be a highly effective, engaging, and valuable learning tool.

#### Thematic analysis of free-text responses

Three free-text questions were included to gather additional feedback from the students. A thematic analysis was performed,^30^ and the summarized results are shown below for each question with the percentage of students who gave this response shown in parentheses.

*Q1. What were the best aspects of the chemotaxis simulation?*

1. Time efficiency and speed control (52%)

Students highlighted the ability to speed up time as a useful aspect of the simulation. This feature allowed them to visualize results quickly, making the learning process more efficient compared with a real-time physical laboratory.

2. Increased interest and engagement (48%)

Students found that the virtual laboratory setting made the content more engaging and enjoyable. The interactive nature of the simulation captured their attention, and made the learning more dynamic than reading or passive observation. The visual and interactive elements kept them engaged, with some expressing this approach was a refreshing alternative to a traditional laboratory experiment.

3. Ease of use (44%)

Students frequently mentioned the simplicity and intuitive nature of the simulation. The clear instructions, easy navigation, and simple controls made the simulation accessible, allowing them to focus on learning rather than dealing with challenging-to-use software.

4. Visual and interactive learning (40%)

Students appreciated the visual representation of neutrophil migration and the ability to manipulate and observe cells’ movements. This hands-on interaction and graphical display helped reinforce understanding of complex biological concepts, making the simulation more engaging and memorable.

5. Improved understanding of concepts (36%)

The simulation helped the students better understand the principles of chemotaxis. The ability to visualize cell movements in real-time and test different chemoattractants helped solidify their understanding. The students found it valuable for breaking down complex ideas through a visual learning experience.

6. Error-free exploration and repetition (28%)

The ability to repeat the simulation without worrying about mistakes was a major highlight for students. This feature allowed them to practice and experiment freely, promoting deeper understanding without the pressure of generating “perfect” data on the first attempt. It should be noted, students could submit their data after multiple attempts of using the software.

7. Measurement and accuracy (20%)

The simulation allowed for accurate measurement of migration, such as field-of-view magnification zooming to measure distance travelled. This functionality added a level of precision that enhanced their understanding of the chemotaxis process.

8. Preparation for hands-on labs (20%)

While the simulation helped students understand the concepts, they still missed the practical, hands-on aspect of laboratory work. They saw the simulation as a useful precursor to a real lab experience, helping them prepare and setting expectations for what they would encounter. However, they emphasized the value of eventually applying the concepts in a physical setting.

9. Visual learning enhancement (16%)

For students who learn best through visuals, the simulation offered an opportunity to observe abstract biological processes, like neutrophil migration, in a more tangible way. The graphics helped them grasp the process of chemotaxis better than purely a theoretical explanation.

10. Preparation for real lab (12%)

A small subset of students indicated that the simulation helped prepare them for actual lab work by giving them a clear expectation of how chemotaxis works. The virtual experience boosted their confidence and readiness for real-world applications.

*Q2. How could the chemotaxis simulation be improved?*

A limited number of responses (19) were received for this question. In many cases, this question was left blank or the students indicated “none” as their response.

1. Zoom and scrolling functionality (48%)

Some students requested finer control and smoother adjustments when magnifying the field of view and moving the grid. They felt that the lower sensitivity hindered their ability for precise measurement. It should be noted that the required level of measurement accuracy was sufficient to determine any cellular defects that may have existed in the patient samples.

2. No improvements needed (32%)

A significant number of students indicated that the simulation had no suggested improvements or it was “perfect” (12%). These responses reflect satisfaction with the current design, features, and overall functionality.

3. Instructions and clarity (16%)

Some students requested having the instructions embedded within the simulation or making them more prominent. Some found the current instructions confusing or insufficient, particularly regarding the control settings or how to execute specific tasks. It should be noted that most students found the instructions very clear (see Likert data).

4. Measurement tools and accuracy (8%)

A small group of students requested improved measurement tools, such as adding a movable ruler or making the tick marks on the grid more prominent to take precise measurements. As noted above, the degree of precision coded into the simulation is sufficient to determine the immune status of the patient samples, as the simulation was used to generate the reference ranges.

5. Link to case studies (4%)

A small number of students requested more connection to real-world case studies, particularly linking the simulation to immunological theory and patient samples. This could help deepen the theoretical understanding while using the simulation.

*Q3. What were the most challenging aspects?*

1. Distance calculation and measurement (80%)

The most frequently cited challenge was accurately measuring the migration distance of the cells. Students found it difficult to align the grid with the cells or wells, especially due to the sensitivity of the controls or lack of precision. Many mentioned that calculating the distance was challenging due to the grid size or alignment issues with the grid’s boundaries and cell markers.

2. Uncertainty about errors or results (20%)

Some students expressed confusion about whether the variations they observed in their results were due to their own errors or the actual simulation. This uncertainty added to the challenge of interpreting data correctly, especially when results seemed unexpected. It should be noted that this is also true for a traditional F-to-F laboratory exercise.

## Discussion

The integration of a computer-based simulation into an undergraduate immunology course provides an innovative approach to bridge the gap between theoretical concepts and hands-on experimental practice. In this study, we developed a simulation that models neutrophil chemotaxis, enabling students to engage with complex immunological processes in a safe, accessible, and pedagogically flexible environment.

This simulation-based approach offers several educational advantages that enhance both engagement and learning outcomes. By requiring students to actively participate in loading reagents, cells, and observing neutrophil migration patterns, they are more actively engaged in learning and, importantly, learning about a clinically relevant assay that is otherwise inaccessible due to resource and logistical constraints. This not only fosters a deeper conceptual understanding of neutrophil chemotaxis but also encourages the development of critical scientific skills such as hypothesis generation and data interpretation. Our approach aligns with Bloom’s Taxonomy by supporting the progression toward higher-order skills.^35^ The simulation also aligns with constructivist approaches by allowing students to build knowledge through active experimentation and reflection. The simulation moves students beyond simple recall of information toward higher-order thinking tasks, including application, analysis, and evaluation. For example, comparing normal versus patient sample responses, using reference ranges, or explaining abnormal results with control samples encourages analytical reasoning. Students also routinely commented on the ability to repeat the simulation without penalty, suggesting increased psychological safety, a factor that is linked to deeper learning.^36^

Online educational resources also help address disparities in access to laboratory experiences. Institutions without Biological Safety Level 2 facilities may be unable to offer direct work with human-derived neutrophils. Our approach mitigates this limitation by providing a safe, scalable alternative that can be implemented in both in-person and remote learning contexts. This supports educational equity by ensuring all students, regardless of institutional resources, have access to high-quality immunology training. Last, by integrating case studies with clinical context, this approach can further enhance the relevance of simulations to medical and biomedical curricula. This bridges the gap between theoretical knowledge and clinical application, preparing students for future professional roles in healthcare and research.

While the focus of our approach related to neutrophil migration defects associated with immune deficiency diseases, other events can also lead to changes in neutrophil chemotaxis, including severe infection,^37^ sepsis,^38^ and cancer.^39^ Hence, with some minor modification to the interface, customizable parameters could be integrated to reflect disease-specific variations in chemotactic behavior, such as is seen with leukocyte-adhesion deficiency.^32^^,^^40^

### Limitations

As with any intervention, there are limitations with this approach. The number of students who submitted a formal report was moderate (24), which may affect the impact of our approach on written performance. However, we were still able to collect qualitative feedback from all students as they were required to submit their report for formative feedback. We did not have a distinct control group, where students did not experience the simulation. However, receiving ethical clearance for a no-intervention control group can be challenging at many institutions. Computer simulations cannot, however, provide direct hands-on experience in handling neutrophils or using chemotaxis chambers, which are crucial components of laboratory training. This speaks to the potential development of hand skills and transferability of learned skills in the simulation to the real-world diagnostic setting. At present, we do not foresee this to be an option with this exact technique as the cost to purchase the required microscopes is prohibitive. Hence, simulations should, where possible, be used as a complement rather than a replacement for practical laboratory experience. Recent reports have demonstrated that the provision of tactile authenticity can bridge the gap between online and hands-on learning.^41^ Hybrid teaching models that combine simulations with wet-lab sessions offer a balanced approach.^6^^,^^27^^,^^42^ While other approaches could be used, such as the Boyden chamber, the issues with true patient samples and reproducibility still exist. One approach could be the use of cell lines, but they may not mirror the exact response seen with primary cells and, unless they are made deficient in a receptor expression, the defects can be challenging to demonstrate.

### Future work

In the future, when considering the current approach, we will include clinical case notes for the students to understand potential issues commonly associated with defects in neutrophil chemotaxis. An example of a potential case study that could be used is provided in the Supplementary materials. We could also provide students with the opportunity to test varying concentrations of chemoattractants and how this affects neutrophil migration distances. Last, we intend to develop additional simulations that replicate other neutrophil immune deficiencies, such as adherence defects.^43^ We may also offer students the opportunity to test different doses of each chemoattractant, measure distances travelled, and plot the data. Similarly, the effect of time on distance migration could be readily assessed,^37^ as well as measured changes in 4 chemotactic function indicators, including chemotaxis distance, chemotaxis cell ratio, chemotaxis index, and maximum speed of chemotaxis. These parameters could also be added to our simulation to provide even more detailed analysis of changes in neutrophil functions.

### Conclusion

Overall, this simulation provides a flexible and accessible platform for teaching neutrophil chemotaxis. It allows students to explore key immunological concepts in a safe, cost-effective environment, and lays the foundation for more sophisticated virtual or hybrid learning experiences. By integrating clinical data and allowing for the manipulation of multiple experimental variables, the simulation can significantly enhance student understanding of immune cell behavior in health and disease. More broadly, this approach could be readily applied to other areas of immunology or medical education where access to patient samples is not possible in a university environment.

## Supplementary Material

vlaf049_Supplementary_Data

## Data Availability

To access the data that support the findings of this study please contact the corresponding author via email.
