# Supplementary material for: Teaching the laboratory assessment of neutrophil chemotaxis: a simulation-based approach for undergraduate immunology
Source: Immunohorizons. 2025 Oct 9;9(11):vlaf049. doi: 10.1093/immhor/vlaf049 (PMC12597877; doi:10.1093/immhor/vlaf049)
Supplement: vlaf049_Supplementary_Data [file vlaf049_supplementary_data.zip › Combined Supplementary Materials.pdf]

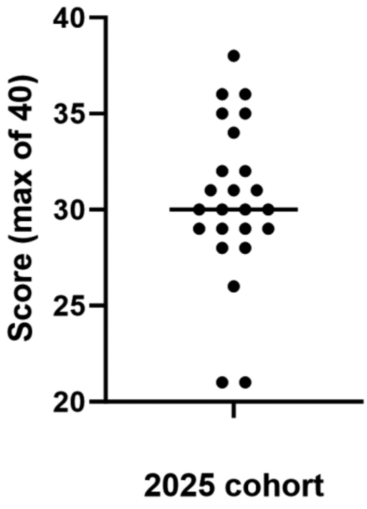

Figure S1

Virtual Neutrophil Chemotaxis

This computer practical is aimed at demonstrating how the movement of purified neutrophils towards a chemical gradient (chemotaxis) is assessed in a clinical Immunology laboratory. Not having access to specialist microscopes, true patient samples and a lengthy incubation means that this practical cannot be performed F-2-F. Hence, we have developed a simulation to teach you the fundamental principles and illustrate how defects in adhesion molecule expression can affect neutrophil migration.

In the laboratory, neutrophils are first isolated from the peripheral blood of a normal control and patient and then diluted to the required density. An agar filled petri dish with three equally spaced wells are used for the assay. Neutrophils are loaded into the central well with diluent loaded into the left well and a chemoattractant into the right. Chemoattractants vary, and include: bacterial tripeptide, fMLP (formylated methionine-leucine-phenylalanine), PMA (phorbol myristate acetate), LPS (lipopolysaccharide), C5a (Complement protein 5a) and LTB_4 (Leukotriene B_4). The chemoattractant diffuses out of the well creating a concentration gradient. Over a 2-hour incubation period at 37°C, some neutrophils will migrate towards the diluent (random migration) or migrate "up" the concentration gradient towards the chemoattractant (directed chemotaxis). The distance migrated after 2-hours is then measured using a microscope eyepiece with a defined grid pattern. The distance migrated is then compared to normal reference ranges to identify any deficiencies.

In this practical, you will test the ability of virtual neutrophils isolated from a normal control and two patient samples against two different chemoattractants. The distance migrated will then be compared to a normal reference range to determine if any defects exist in the control or patient samples.

Aims

1: Understand the basic principles of chemotaxis.

2: Understand how chemotaxis is performed in a laboratory setting.

3: Determine the immunological status of two patient samples using a supplied reference range.

1. To access the Virtual chemotaxis visit https://garethdenyer.github.io/Chemotaxis/

   Note, the address is CaSe sensitive.

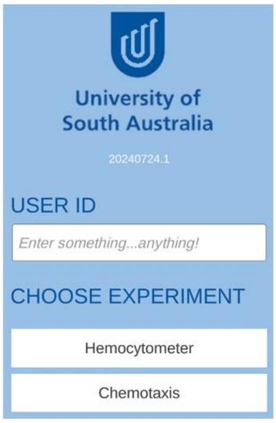

Fig S2. Front page of the chemotaxis simulation.

2. Enter the last 4-digits of your student ID and select the rectangle titled "Chemotaxis" (Fig S2).
3. After a short delay, the virtual laboratory environment will appear.

4. Using the provided Student allocation sheet, locate your Student ID and which **2** Patient samples you have been allocated (called A, B or C). Make a note of which chemoattractant should be tested for each patient sample (i.e., fMLP, PMA, LPS, C5a or LTB_4).

5. On startup, the screen will show a low-power view of the chemotaxis plate (Fig S3). The three wells will be visible (as highlighted by the yellow circles). The central well is where the control or patient cells will be added (in separate plates), the left well is where diluent will be added, and the right well is where the chemoattractant is loaded.

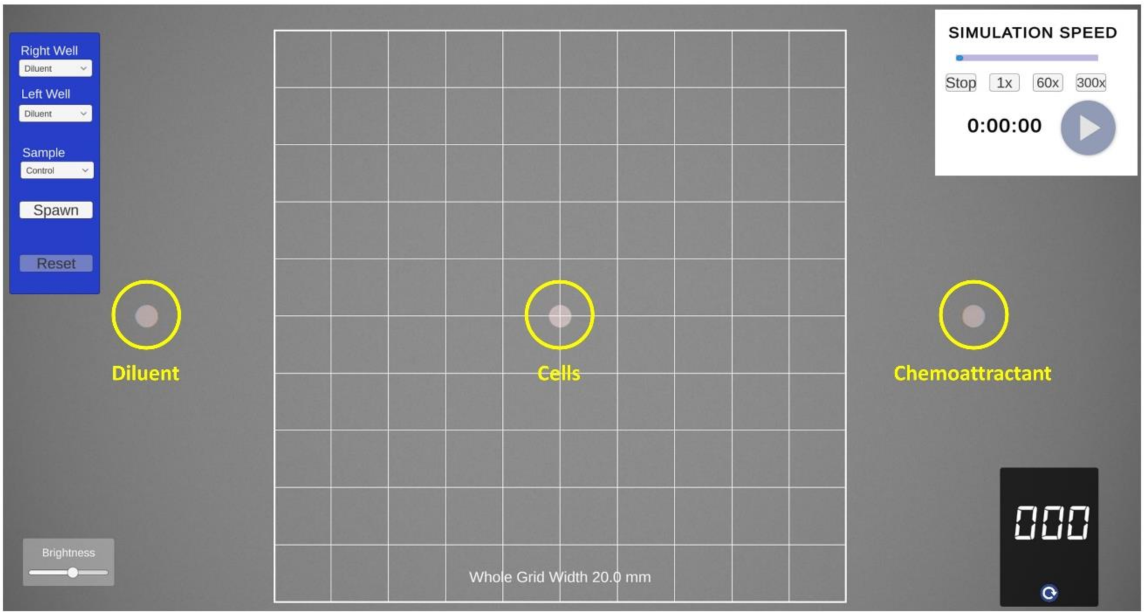

Fig S3. Low power view of the chemotaxis plate on startup. The left, central and right well are shown by the yellow circles. **Note,** these circles and labels will **not** appear in the simulation.

6. At the bottom left of the screen is the brightness control (Fig S4). This can be adjusted as required.

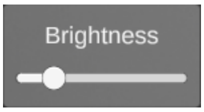

Fig S4. Brightness control slider. (Move right to increase the brightness)

7. On the top left is the panel where reagents can be automatically added to the right and left well.

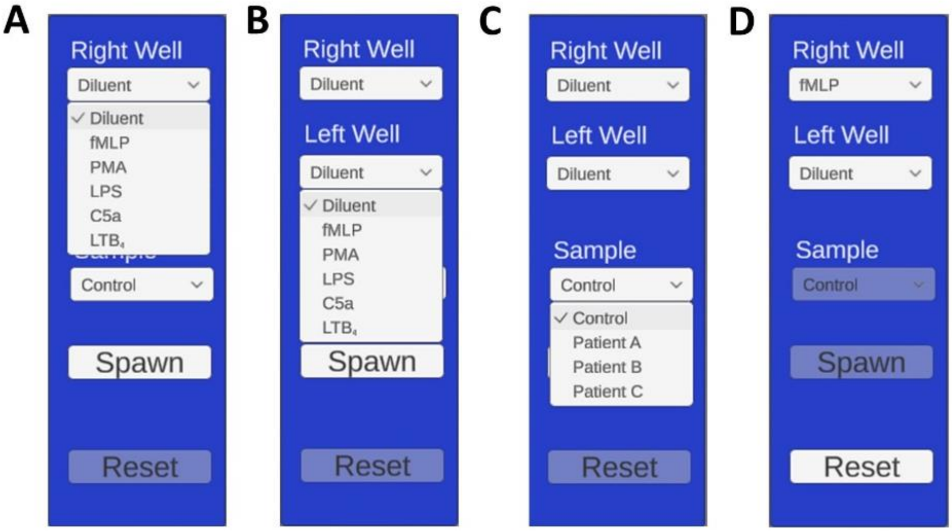

Figure S5. Panel for locating the chemoattractants and control or patient samples.

8. Press the right well "drop down" menu to select the chemoattractant to be used (Fig S5A); then select the left well "drop down" menu and select diluent (Fig S5B); Next, **begin** by selecting the **Control** sample (Fig S5C); once all are selected press the "Spawn" button to load the respective wells (Fig S5D).
9. If you make a mistake, press the "Reset" button to start again.
10. Using the mouse scroll wheel, ZOOM into the central well. You should see the central well filled with neutrophils (red cells) (Fig S6).

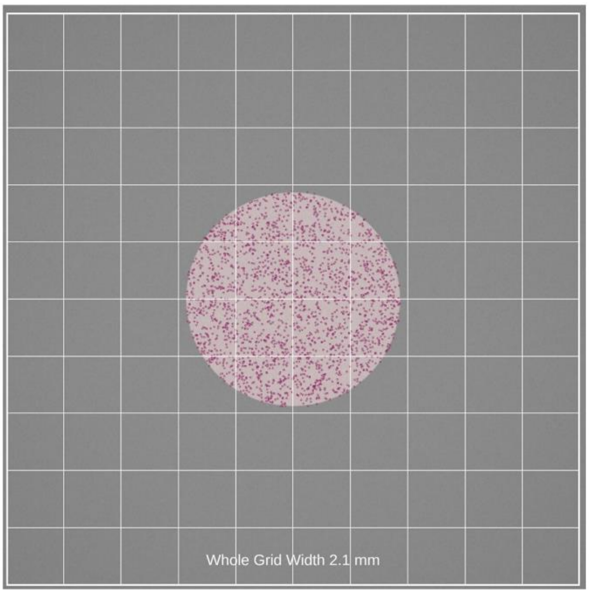

Fig S6. Zoomed view of the central well. Neutrophils (red cells) can be clearly seen contained within the well.

Note the scale at the bottom of the grid. This grid will be used to measure the distance travelled by the cells after incubation.

11. In the top right-hand corner of the screen, you will see a timer which will **START** the incubation (Fig S7A). Initially select the 300x button and then press the arrow icon (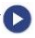) to begin. The timer will begin counting, and you should incubate for ~**2 hours**. To get closer to this time, you can change the speed to 60x or 1x, however it does **NOT** need to be exactly 2 hours (Fig S7B).

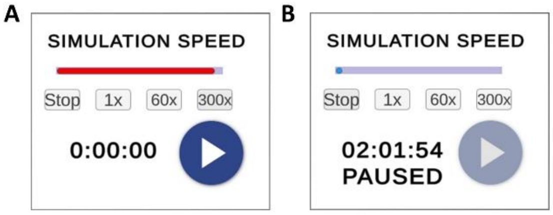

Fig S7. Button to START the simulation and control the speed of incubation.

12. After 2 hours, some cells will have migrated toward the left well containing the diluent (random migration), while the majority should have migrated towards the right well containing the chemoattractant (Fig S8).

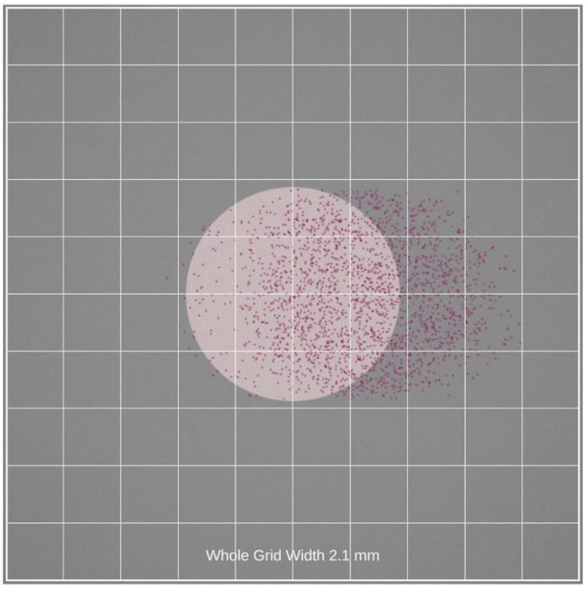

Fig S8. View of the chemotaxis plate after ~2 hours incubation. Note some cells have migrated toward the diluent (left well), while most have migrated toward the chemoattractant (right well).

13. First, measure the random migration. Using the mouse scroll wheel, slowly ZOOM in to view the cells that have migrated towards the diluent (left direction) (Fig S9).

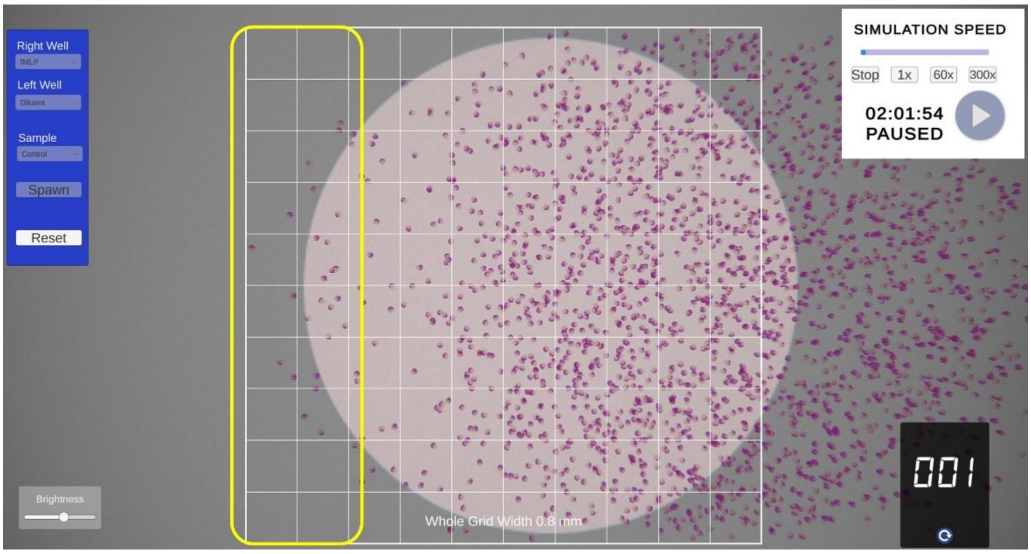

Fig S9. Magnified view of the central well showing the small random migration of cells towards the left well (diluent) (yellow rectangle).

14. In the laboratory, the grid shown is located within the microscope eyepiece. It allows you to determine the distance that the cells have migrated.

15. Using the arrow keys (Fig S10), position the 10 x 10 grid so that the distance migrated can be captured by the grid.

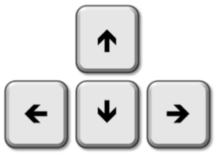

Fig S10. The keyboard arrow keys can be used to move the grid so that the distance of migration of the cells can be easily determined.

16. In the example shown in Fig S11, the total width of the whole 10 square grid is 0.8mm, hence each individual square represents 0.08mm. The cells have migrated approximately 1 grid away from the central well, so this should be recorded as ~0.08mm (random migration).

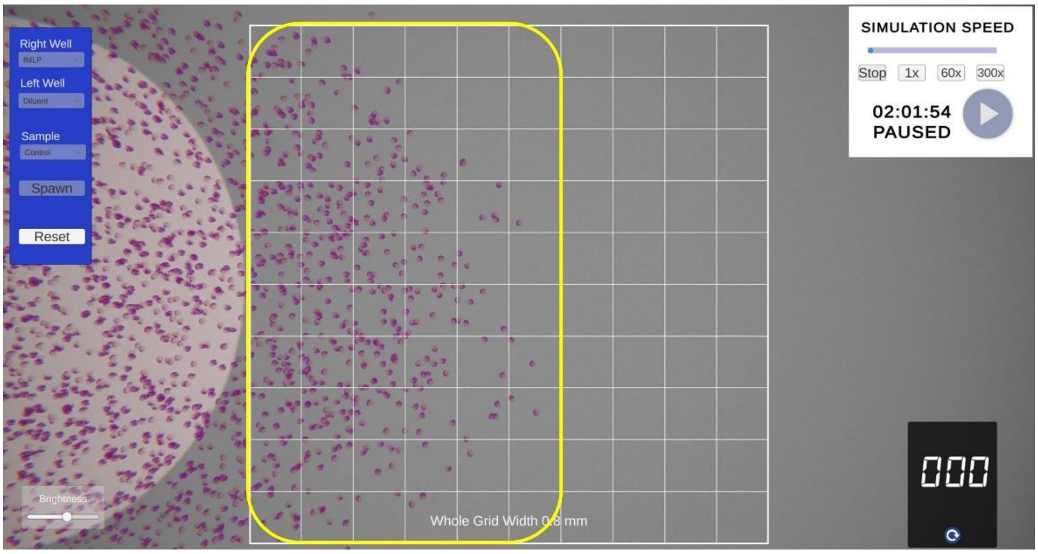

Fig S11. The grid has now been moved to the right hand well and the maximum distance cells have migrated toward the right well (chemoattractant) has been captured (yellow rectangle).

17. You should place the left-hand side of the grid so that it touches the right-hand side edge of the well (Fig S11).

18. You can then count how many squares the cells have migrated. In the example in Fig S11, this is approximately 6 squares, with each square representing 0.08mm, so the total distance is calculated as: 6 x 0.08mm = 0.48mm. If a cell is mid-way between a grid, you should zoom in further.

19. The size of the scale will adjust by using the scroll wheel and the spacing will change accordingly. ***Take note of this before calculating the total distance migrated by the cells***.

20. If cells have migrated further than the grid width, select a cell which is found in the far right of the grid, click on it, and it will change to green to indicate it has been selected (Fig S12).

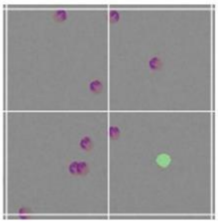

Fig S12. The green dot represents a cell that has migrated near the far right of the grid. To help with measuring further travel it has been selected, as shown by the green colour. You can now move the grid without losing your position and hence measurement.

21. Once this has been completed for the **Control** sample, record **both** the random and directed chemotaxis results in Table S1.

22. Now press the reset button (Reset) and repeat the assay for the two patient samples allocated to you (A, B or C).

23. Once all results have been recorded, compare your results to the provide reference range (Table S2) to determine if the results are valid for the control and if any defects exist in the patient samples.

Table S1. Results recording.

**Cells**	**Chemoattractant**	**Random migration (mm)**	**Directed migration (mm)**
Control 1			
Control 2			
Patient A			
Patient B			
Patient C			

Note, for the cell which has not been allocated to you, leave the corresponding well empty.

Table S2. Reference ranges for data interpretation.

**Chemoattractant**	**Normal random migration (mm)**	**Normal chemotaxis (mm)**
Diluent	< 0.4	<0.4
fMLP	< 0.4	>3.5
PMA	< 0.4	>2.2
LPS	< 0.4	>2.5
C5a	< 0.4	>2.4
LTB_4	< 0.4	>2.6

Study questions.

1. If cells taken from a normal control migrated less that the normal reference range, could you interpret the patient results? Explain your answer.
2. Considering the results for the normal control, discuss the results collected for your two patient samples.
3. If the patient cells were found to migrate less than the reference range, give **one** immunological explanation for this result? (***Hint***, look at the leukocyte migration & inflammation lecture).
4. Briefly explain the nature of the two chemoattractants assigned to you and why they are suitable to be used in this assay.
5. What would be the effect(s) in the body if neutrophils could not effectively perform chemotaxis?

Clinical Case Notes: Suspected Neutrophil Chemotaxis Defect

Patient Information

Patient: Sarah M., 8-year-old female
Date of Admission: March 15, 2023
Chief Complaint: Recurrent skin infections and delayed wound healing

Presenting History

Sarah presents with a 6-month history of recurrent bacterial skin infections that have been increasingly difficult to treat with standard antibiotic therapy. Her parents report that minor cuts and abrasions take weeks to heal and often become infected despite proper wound care.

Clinical History

Current Episode

- **Onset:** 5 days ago, developed cellulitis on left forearm following a minor scratch from playground equipment
- **Progression:** Despite oral antibiotics (amoxicillin-clavulanate), the infection has spread and become more erythematous
- **Associated symptoms:** Low-grade fever (38.2°C), localised warmth and swelling

Past Medical History

- **Recurrent infections since age 5:**
  - 8 episodes of bacterial skin infections in past 18 months
  - 3 episodes of pneumonia requiring hospitalisation
  - 2 episodes of otitis media with complications
  - 1 episode of dental abscess
- **Causative organisms:** Predominantly gram-positive bacteria including *Staphylococcus aureus* and *Streptococcus pyogenes*
- **Treatment response:** Infections typically require prolonged antibiotic courses and often progress despite appropriate therapy

Family History

- No family history of immunodeficiency disorders
- Parents and siblings have normal infection patterns
- No consanguinity

Physical Examination

General Appearance

- Well-developed child appearing mildly ill
- Alert and cooperative but fatigued

Vital Signs

- Temperature: 38.2°C
- Heart rate: 95 bpm
- Respiratory rate: 22/min
- Blood pressure: 105/65 mmHg

Skin Examination

- **Left forearm:** 8cm x 5cm area of erythema, warmth, and induration
- **Multiple healing lesions:** Various stages of healing from previous infections
- **Delayed wound healing:** Several minor cuts showing poor healing progression
- **No lymphadenopathy**

Other Systems

- Cardiovascular: Normal heart sounds, no murmurs
- Respiratory: Clear lung fields bilaterally
- Abdomen: Soft, non-tender, no organomegaly

Laboratory Investigations

Complete Blood Count

- **White Blood Cell Count:** 18,500/µL (elevated)
- **Neutrophils:** 14,800/µL (80%) - markedly elevated
- **Lymphocytes:** 2,220/µL (12%)
- **Monocytes:** 1,110/µL (6%)
- **Eosinophils:** 370/µL (2%)
- **Hemoglobin:** 11.8 g/dL
- **Platelets:** 425,000/µL

Basic Metabolic Panel

- Within normal limits

Inflammatory Markers

- **C-reactive protein:** 45 mg/L (elevated; normal <3 mg/L)
- **Erythrocyte sedimentation rate:** 55 mm/hr (elevated)

Microbiological Studies

- **Wound culture:** *Staphylococcus aureus* (methicillin-sensitive)
- **Blood cultures:** Pending

Immunological Assessment

Basic Immunoglobulin Levels

- **IgG:** 1,200 mg/dL (normal for age)
- **IgA:** 180 mg/dL (normal for age)
- **IgM:** 95 mg/dL (normal for age)

Complement Studies

- **CH50:** Normal
- **C3, C4:** Normal levels

Preliminary Neutrophil Function Studies

- **Neutrophil count:** Adequate numbers present
- **Morphology:** Normal appearance on blood smear
- **Nitroblue tetrazolium (NBT) test:** Normal (rules out chronic granulomatous disease)

Clinical Reasoning and Differential Diagnosis

Primary Consideration: Neutrophil Chemotaxis Defect

The pattern of recurrent bacterial infections, particularly involving skin and soft tissues, combined with normal neutrophil numbers but poor infection control, suggests a functional neutrophil defect. The specific pattern points toward a chemotaxis disorder.

Supporting evidence:

- Recurrent infections with encapsulated bacteria
- Normal neutrophil counts during infections
- Delayed wound healing
- Poor response to appropriate antibiotic therapy
- Normal immunoglobulin levels ruling out humoral defects

Differential Diagnoses Considered

1. **Chronic Granulomatous Disease:** Ruled out by normal NBT test
2. **Leukocyte Adhesion Deficiency:** Would expect more severe presentation and higher white counts
3. **Hyper-IgE Syndrome:** No eczema, elevated IgE, or characteristic facial features
4. **Common Variable Immunodeficiency:** Normal immunoglobulin levels make this unlikely

Proposed Diagnostic Test: Under-Agarose Chemotaxis Assay

Rationale for Testing

Given the clinical presentation suggestive of neutrophil chemotaxis defect, the under-agarose method is the most appropriate diagnostic test to assess neutrophil migration in response to chemoattractants.

Test Methodology

The under-agarose assay measures neutrophil chemotaxis by:

1. Placing patient neutrophils in wells cut in agarose gel
2. Adding chemoattractant (such as N-formyl-methionyl-leucyl-phenylalanine or complement C5a) to adjacent wells
3. Measuring the distance neutrophils migrate through the agarose toward the chemoattractant over a specified time period
4. Comparing results to healthy control neutrophils

Expected Results

If neutrophil chemotaxis defect is present:

- **Reduced migration distance** compared to healthy controls
- **Normal random migration** (ruling out general motility defects)
- **Specific defect in directed migration** toward chemoattractants

Treatment Plan

Immediate Management

1. **Antibiotic therapy:** Switch to IV clindamycin based on culture sensitivities
2. **Wound care:** Aggressive local wound management
3. **Supportive care:** Analgesics and antipyretics as needed

Long-term Management (if chemotaxis defect confirmed)

1. **Prophylactic antibiotics:** Consider trimethoprim-sulfamethoxazole
2. **Aggressive wound care:** Immediate attention to any skin breaks
3. **Patient/family education:** Recognition of early infection signs
4. **Regular monitoring:** Periodic assessment for complications
5. **Vaccination:** Ensure up-to-date with all recommended vaccines

Educational Points for Immunology Students

Neutrophil Chemotaxis

Chemotaxis is the directed migration of neutrophils toward sites of infection or inflammation in response to chemical gradients. This process involves:

- **Recognition** of chemoattractants via surface receptors
- **Signal transduction** leading to cytoskeletal rearrangement
- **Directed migration** through tissues toward the infection site

Clinical Significance

Defects in neutrophil chemotaxis result in:

- Inability of neutrophils to reach infection sites effectively
- Persistent bacterial infections despite adequate neutrophil numbers
- Poor wound healing due to impaired inflammatory response
- Predominance of bacterial (especially gram-positive) infections

Diagnostic Considerations

The under-agarose method specifically tests:

- **Chemotaxis:** Directed migration toward chemoattractants
- **Chemokinesis:** Random migration (control measurement)
- **Comparison with controls:** Essential for interpretation

This case illustrates how normal neutrophil numbers do not guarantee normal neutrophil function, emphasising the importance of functional assays in evaluating recurrent infections.

Dear Student

Measuring the ability of neutrophils to migrate (chemotaxis) is important to determine neutrophil activity in a clinical immunology laboratory. However, we do not have the required equipment to perform this assay Face-2-Face. Hence, we have developed a computer simulation to teach you the basic principles. I am keen to learn how well the chemotaxis simulation helped you in this understanding. This short questionnaire will anonymously collect your feedback. Please circle the response which matches most closely with your experiences or add text responses in the latter questions. Completing this questionnaire should only take **5 minutes**. Thank you for your participation.

1: The simulation enhanced my learning the **principles** of chemotaxis and made the concepts clear to me

Strongly agree Agree Neutral Disagree Strongly disagree

2: I found the chemotaxis simulation **easy** to use.

Strongly agree Agree Neutral Disagree Strongly disagree

3: The **graphics** appeared clear and understandable making the learning morerealistic.

Strongly agree Agree Neutral Disagree Strongly disagree

4: The written instructions were **clear** and easy to follow.

Strongly agree Agree Neutral Disagree Strongly disagree

5: **Before** the simulation, my understanding of a chemotaxis assay was:

Very good Good Neutral Poor None

6: **After** the simulation, my understanding of a chemotaxis assay was:

Very good Good Neutral Poor None

7: Did generating a virtual laboratory setting make the content **MORE** or **LESS** interesting?

MORE NEUTRAL LESS

Please briefly explain your answer.

8. After completing the simulation, how confident do you feel in applying the concepts of chemotaxis to a real-world laboratory?

Very confident Confident Neutral Slightly confident Not confident

9. Would you recommend this simulation to other students learning about chemotaxis?

Strongly agree Agree Neutral Disagree Strongly disagree

10: What were the best aspects of the Chemotaxis simulation?

11: How could the chemotaxis simulation be improved?

12: What aspects did you find most challenging (briefly explain why)?

Table S3. Descriptive statistics for student questionnaire Likert scale items.

Parameter	Qu 1	Qu 2	Qu 3	Qu 4	Qu 5	Qu 6	Qu 7	Qu 8	Qu 9
N	67	67	67	67	67	67	67	65	65
Min	3.000	3.000	4.000	3.000	1.000	3.000	3.000	3.000	3.000
25%	4.000	4.000	4.000	4.000	2.000	4.000	5.000	4.000	4.000
Median	5.000	5.000	5.000	5.000	3.000	4.000	5.000	4.000	5.000
75%	5.000	5.000	5.000	5.000	3.000	5.000	5.000	4.000	5.000
Max	5.000	5.000	5.000	5.000	5.000	5.000	5.000	5.000	5.000
Mean	4.507	4.627	4.567	4.582	2.896	4.284	4.836	3.969	4.569
SD	0.5329	0.5730	0.4992	0.5265	0.8373	0.4864	0.4798	0.4319	0.5294
SEM	0.065	0.07	0.061	0.064	0.102	0.059	0.058	0.053	0.065

Table S4. Laboratory report marking rubric.

Criteria	HD (85–100%)	D (75–84%)	C (65–74%)	P (50–64%)	F (<50%)
**Data Accuracy & Recording**	All results recorded accurately; correct units & comparison with reference ranges clearly shown.	Minor errors in measurement or units; correctly interpreted against reference ranges.	Most values are accurate; minor mistakes in comparison or units.	Some values missing or unclear; basic interpretation attempted.	Major errors or omissions in data; incorrect or no comparison to reference values.
**Interpretation of Control & Patient Samples**	Excellent understanding of control validity, patient results, interpretation; clear, logical justification provided.	Good interpretation with valid reasoning & mostly correct analysis.	Adequate understanding with minor gaps or generalised reasoning.	Basic attempt to interpret data with limited explanation.	Incorrect or no interpretation; fails to recognise the role of controls.
**Immunological Reasoning**	Detailed & accurate immunological explanation for defective chemotaxis, referencing relevant immune diseases (e.g. LAD).	Mostly accurate immunological rationale with some detail.	Gives a reasonable explanation with minor inaccuracies or vagueness.	Answer shows limited understanding of immunological mechanisms.	Incorrect or no explanation; lacks immunological insight.
**Chemoattractant Knowledge**	Clearly explains both chemoattractants with accurate scientific detail & relevance to assay use.	Good explanation of both chemoattractants with minor gaps in relevance or depth.	Adequate explanation; some inaccuracies or missing rationale for suitability.	Basic knowledge shown; relevance to the assay not well explained.	Inaccurate, incomplete or missing descriptions of chemoattractants.
**Application to Clinical Context**	Clear response on consequences of defective chemotaxis; strong links to immune function & implications.	Clear & relevant response; links to immune function are present.	Reasonable response with basic understanding of clinical implications.	Generalised answer; limited link to clinical relevance.	No or incorrect response; lacks clinical application.

Criteria	HD (85–100%)	D (75–84%)	C (65–74%)	P (50–64%)	F (<50%)
**Clarity & Scientific Communication**	Clearly structured, concise, uses correct terminology, & free from spelling & grammatical errors.	Well-structured & mostly clear; minor language or structure issues.	Acceptable clarity with some grammar/spelling/structure problems.	Readable but contains frequent errors or poorly structured responses.	Poorly written, unclear, or difficult to follow.
